# Higher-order structural organization of mitochondrial metabolism

**DOI:** 10.1016/j.jbc.2026.113174

**Published:** 2026-05-20

**Authors:** Greg Kafetzopoulos, Jonas Döbler, Panagiotis L. Kastritis

**Affiliations:** 1 Institute of Chemical Biology, National Hellenic Research Foundation, Athens, Greece; 2 Department of Integrative Structural Biochemistry, Institute of Biochemistry and Biotechnology, Martin Luther University Halle-Wittenberg, Halle/Saale, Germany; 3 Biozentrum, Martin Luther University Halle-Wittenberg, Halle/Saale, Germany; 4 Interdisciplinary Research Center HALOmem, Charles Tanford Protein Center, Martin Luther University Halle-Wittenberg, Halle/Saale, Germany

**Keywords:** structural and molecular biology, supramolecular assemblies, mitochondrial metabolons, dynamic protein complexes, scaffolding, filamentous enzyme regulation, conformational landscape, lipid–protein interactions, metabolic modularity, integrative structural modeling

## Abstract

The mitochondrion embeds numerous metabolic, biosynthetic, and signaling pathways occurring within its specialized compartments that collectively define eukaryotic biology. To visualize these, cryogenic electron microscopy (cryo-EM) is now utilized, particularly for resolving biomolecular complexes that were notoriously hard to study with other structural methods. Our review synthesizes recent cryo-EM-based discoveries, analyzes structures related to mitochondrial metabolism, and explains related functional aspects of core energy metabolism, biosynthetic processes, transport, and regulatory systems. We report on recent data on mitochondrial enzyme filamentation, higher-order metabolons, enzyme-gating mechanisms, and contact-site nanostructures. In addition to single-particle cryo-EM results, we discuss emerging *in situ* cryo-tomography data, the integration of traditional structural biology approaches, and the use of *in silico* models for less-studied pathways. By mapping hundreds of recent mitochondrial structures, we provide a roadmap that connects structural biochemistry with cell physiology, disclosing the molecular basis of mitochondrial disease. Our collected resources may guide future integrative research aiming to elucidate mitochondrial architecture and function across organisms and conditions.

Mitochondria were first visualized more than 160 years ago. They were originally described as granules in muscle in 1857 (1). Later, they were dubbed as bioblasts (2) and in 1898, their current term was coined (3). The word mitochondria originates from the combination of μίτος and χονδρίον (mitos and chondrion), meaning thread and granule, describing their appearance. Subsequently, Lehninger (among others, reviewed by Potter (4)) contributed to describing their role in energy conversion and oxidative phosphorylation (OXPHOS) (5). Since then, an ever-increasing number of functions and pathways have been associated with these organelles. Advanced studies on mitochondrial functions have increasingly revealed important insights into this organelle’s central role in cellular physiology (6, 7).

Mitochondria support and regulate the energetic and metabolic demands of eukaryotic cells. Part of their role is sensing and responding to environmental signals (8, 9). Furthermore, defects in mitochondrial function are associated with cancer phenotypes (10). They embed major biochemical processes interconnected by a complex web of interactions, the extent of which is still being uncovered (11, 12). These processes are recorded and enriched in specialized databases, such as the MitoCarta database (13). Examples include energy conversion through OXPHOS, lipid and amino acid metabolism, mitochondrial-specific protein synthesis, Ca^++^ buffering, and the initiation of apoptotic signaling (14). In addition, mechanisms for (bio)molecular transport preserve balanced mitochondrial function and continuous communication with other cellular compartments.

Mitochondrial processes are partitioned across distinct mitochondrial compartments, *that is*, the outer membrane, the intermembrane space, the inner membrane, and the matrix. Coordination across these is pivotal for cellular homeostasis and thus regulated at different levels. For example, the intermembrane space has been associated with stress response regulation (15). Sub-compartmentalization within mitochondria is complex because of (a) the presence of a membrane system and (b) high protein concentrations. Concentrations in the matrix are close to ∼300 to 500 mg/ml, which correspond to ∼5 to 10 mM for a ∼50 kDa average protein size, rivaling those within a protein crystal (16). Therefore, macromolecular crowding within the densely packed mitochondria influences diffusion mechanisms and, consequently, all relevant physical–chemical properties (*e.g.,* biochemical reaction rates, signaling events, stress response) (17). Such a dense crowded environment makes understanding mitochondrial function ever more challenging. Latest studies on mitochondrial proteomics suggest an average participation of a mitochondrial protein in at least six higher-order assemblies (18).

Certainly, such an environment can only be studied through analysis of its structure at the molecular level. Historically, X-ray crystallography and nuclear magnetic resonance (NMR) spectroscopy have been used to study individual mitochondrial proteins. For example, those methods elucidated ultra-high-resolution features of the structure of pyruvate dehydrogenase and the dynamics of lipoyl arms in α-keto acid dehydrogenases (19). However, these methods require large amounts of concentrated sample to either form crystals or generate enough signal for NMR. Many native complexes, particularly membrane-embedded biomolecular complexes and dynamic, transient, multi-subunit machines, termed metabolons (20), remained structurally uncharacterized due to their sheer size, flexibility, and heterogeneity. Cryo-EM has overcome many of these limitations, providing data for the structural analysis of macromolecular assemblies in a closer-to-life state (*i.e.,* in a more direct depiction of their biologic processes and functions) and in multiple functional conformations, *e.g.,* for the inner structure of the pyruvate dehydrogenase complex (21).

Overall, exciting developments are shifting the field of mitochondrial biology toward a more holistic view, where structure is directly linked to metabolic regulation, signaling pathways, and pathology. In addition, mutations affecting mitochondrial structural assemblies are being recognized in a wide range of organisms, including plants (22, 23), where their phenotypes are directly affected, *e.g.,* becoming resilient to drought (24). Mutations in mitochondrial assemblies also play a prominent role in human disorders (25), including neurodegenerative diseases, cardiomyopathies, and metabolic syndromes (26).

## Direct multi-scale mapping of mitochondrial metabolism by cryo-EM

The core advantages of cryo-EM over traditional structural methods have been thoroughly discussed in the literature (*e.g.,* in (27, 28, 29) [https://www.sciencedirect.com/science/article/pii/S0927796X2600017]) and were acknowledged with the award of the Nobel Prize in Chemistry in 2017 to the developers of the method. Arguably, the most powerful advantage is the direct imaging of the material under study in real space and at high resolution. If real-space images are captured, then a direct understanding of the recorded sample regions is possible. The cryogenic sample that is imaged at an electron microscope can stem from overexpressed sources, but also from native material, including tissues, *e.g.,* from muscle (30). Given that this material includes repeating structural signatures of a given molecule or assembly, averaging algorithms (31) can analyze the recorded images, termed micrographs. This methodology increases the signal-to-noise ratio of the structure under investigation, thereby improving its resolution. If enough particles are retrieved, three-dimensional reconstructions are derived, which can reach atomic resolution.

In a real-space image, such as a photo from a phone, identifying object(s) of interest, deriving relative distances and shapes (conformations) is visually intuitive. In cryo-EM, this is evident in the case of analyzing cellular homogenates or intact cells. Examples are the interactions of enzymes in pyruvate oxidation (32), and the proximity of non-mitochondrial biomolecular complexes with the outer mitochondrial membrane (33, 34). Consequently, it is undeniable that cryo-EM and its associated methods are bound to advance our knowledge, not only for individual molecules and their protein complexes but most importantly for their structures within heterogeneous and increasingly complex specimens.

**Figure 1:**
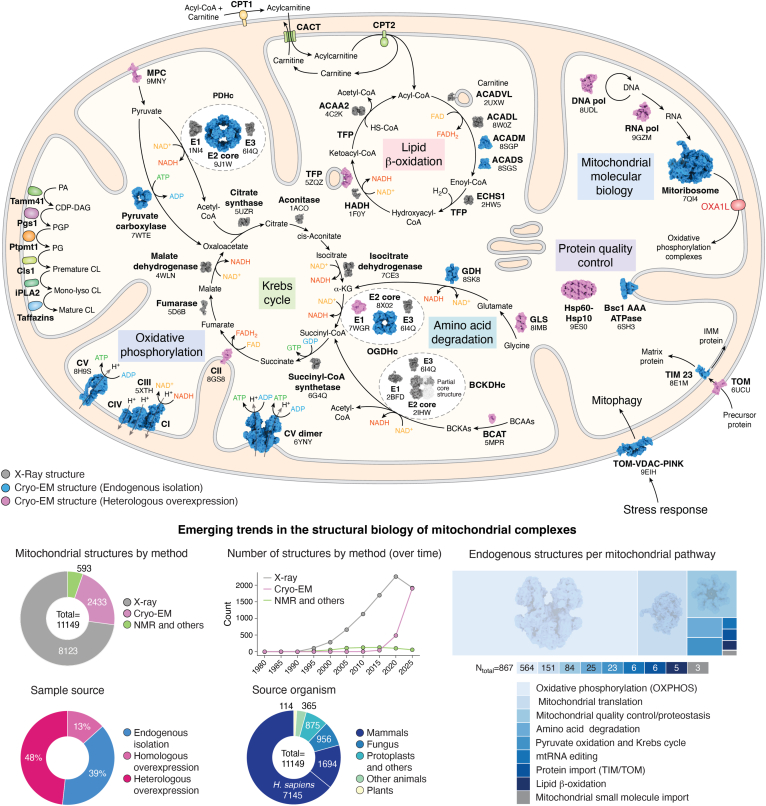
**Structural overview of discussed mitochondrial pathways and overall structural coverage in the protein data bank (PDB).** Schematic representation of major mitochondrial pathways with representative molecular structures in relative scales. Central carbon metabolism begins with mitochondrial pyruvate import through the mitochondrial pyruvate carrier (MPC). Pyruvate is converted to acetyl-CoA by the pyruvate dehydrogenase complex (PDHc) or to oxaloacetate by pyruvate carboxylase, feeding the Krebs cycle where enzymes generate reducing equivalents that drive oxidative phosphorylation. Cryo-EM structures of respiratory complexes reveal multiple catalytic states and the organization of complexes I-IV into supercomplexes, while ATP synthase dimers architecturally organize the cristae membranes and catalyze ATP production. Additional metabolic pathways include amino acid degradation and fatty acid β-oxidation, where acyl-CoA dehydrogenases and the mitochondrial trifunctional protein catalyze sequential oxidation reactions. Cardiolipin biosynthesis contributes to inner-membrane organization and respiratory complex stability. Mitochondrial gene expression produces essential respiratory subunits through replication, transcription, and translation systems that include DNA polymerase γ, mitochondrial RNA polymerase, and the mitoribosome. Most mitochondrial proteins are imported through the TOM and TIM23 translocases, while ATP-dependent chaperones and proteases mediate protein folding, quality control, and stress responses. Panels at the bottom summarize structural statistics across mitochondrial complexes, including structure determination methods, temporal trends, source organisms, and sample preparation strategies.

In this review, we pursue the question: Is cryo-EM currently the leading resource in the effort to map mitochondrial structure(s) and biochemistry*?* To address this, we systematically probed the recent literature concerning the structural biology of mitochondrial metabolism. We uncovered that adding the structural dimension of mitochondrial pathways is being rapidly consolidated in a systems-based manner, almost entirely fueled by cryo-EM (Fig. 1). We constructed the integrative Figure 1 after a simple search in the Protein Data Bank (35) for resolved complexes with mitochondrial-associated GO terms, restricted to experimentally determined structures from Eukaryota. The initial dataset was curated to remove non-mitochondrial or ambiguous entries (*e.g.,* cytosolic ribosomal particles and chloroplast-derived complexes), followed by filtering for cryo-EM structures and, where possible, endogenous or near-endogenous assemblies. For structural representation within pathways shown in Figure 1, we applied consistent selection criteria prioritizing human structures, resolutions better than 4 Å, and endogenous assemblies when accessible.

Overall, we identified 11,149 mitochondrial structures, and their coverage is dominated by X-ray crystallography. However, cryo-EM shows rapid growth, with structures enriched in mammals and concentrated in OXPHOS and mitochondrial translation. 8123 derive from X-ray crystallography and 2433 from cryo-EM, the only method showing exponential growth. Interestingly, 39% of cryo-EM structures originate from endogenous isolations, compared with 48% and 13% from heterologous and homologous sources. Endogenous cryo-EM structures cluster in oxidative phosphorylation and mitochondrial translation, whereas nearly all other pathways remain sparsely sampled. Such trends underscore both advances and outstanding gaps, and broader organismal and pathway coverage will be essential for defining structural principles of mitochondrial function. However, systematically matching the structural data to mitochondrial pathways is now becoming feasible, clearly showing that, indeed, cryo-EM fuels deeper structure–function understanding of mitochondrial biochemistry. Overall, our review illustrates the widespread structural analysis of mitochondrial complexes previously structurally unknown (or resolved in “pieces”) and argues toward three core concepts.

(i) *Spatial organization.* Cryo-EM reveals that mitochondrial biochemistry is spatially organized through specific, transient macromolecular interfaces. Mitochondrial pathways are composed of precisely regulated higher-order flexible assemblies and membrane-associated networks.
(ii) *Structural plasticity.* Cryo-EM frequently demonstrates that structural plasticity and state-dependent conformations are functional states for many mitochondrial complexes, which exist in multiple conformational and oligomeric states.
(iii) *Context-dependent architecture.* Cryo-EM catalyzes a conceptual shift toward endogenous and *in situ* structural biology, embedding mitochondrial structural analysis within integrative frameworks and bringing the once-unthinkable goal of a multiscale virtual cell closer to reality.

**Figure 2:**
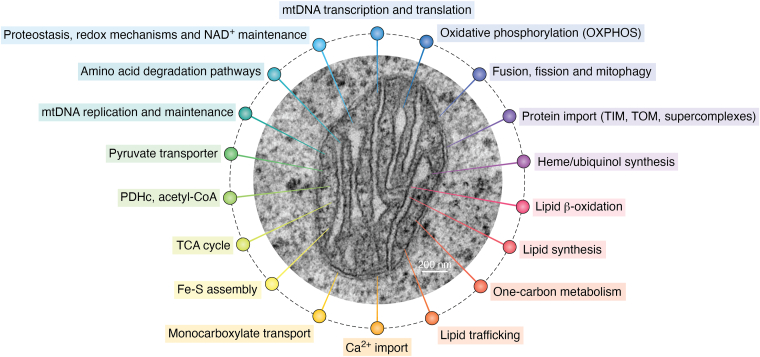
**Mitochondrial pathways described in this review.** A eukaryotic mitochondrion is shown in the middle, and with a different color-code the specific metabolic pathways along with the major localizations of their biomolecular components. The mitochondrion is *from Chaetomium thermophilum* hyphae. Other data from this EM session were published previously (52). Cells were harvested by Dr. Fotis L. Kyrilis (National Hellenic Research Foundation, Greece) and samples were analyzed by Dr. Gerd Hause (Martin Luther University Halle-Wittenberg, Germany).

Below, we provide an extensive overview of mitochondrial macromolecular complexes resolved by cryo-EM. We emphasize progress in the structural analysis of key steps in energy conversion, biosynthetic processes, amino acid metabolism, metabolite transport, gene expression, mitochondrial dynamics, and redox balance (Figs. 1 and 2). Representative structural studies across each of these pathways are highlighted, aiming to depict how cryo-EM contributes to molecular visualization, which in turn provides mechanistic understanding of mitochondrial function across eukaryotes, and, in particular, human health and disease. Throughout our review, we distinguish between high-resolution cryo-EM structures, *in situ* cryo-ET observations, and systems supported by complementary structural or computational approaches.

## Energy conversion and core metabolism

Pyruvate, the end product of glycolysis, is decarboxylated by the pyruvate dehydrogenase complex (PDHc) within the mitochondrial matrix to yield acetyl-CoA, NADH, and CO_2_, in a biochemical sequence of events that requires CoA and NAD^+^. This acetyl-CoA enters the Krebs cycle (tricarboxylic acid (TCA) cycle), along with other loaded CoAs, where it fuels the reduction of NAD^+^ and FAD. NADH and FADH_2_ are electron carriers that transfer high-energy electrons to the electron transport chain (ETC) to establish the electrochemical gradient that powers ATP synthesis *via* the ATP synthase megacomplex. In parallel, mitochondrial β-oxidation of fatty acids is an additional source of acetyl-CoA and reducing equivalents that links lipid catabolism to mitochondrial energy conversion. These pathways constitute a bioenergetic network within the mitochondrial matrix and its inner membrane. It is likely influenced by an underlying multifaceted supramolecular enzyme organization, which is, in turn, drastically affected by cellular metabolic states and energy needs.

### Pyruvate dehydrogenase complex (PDHc) and acetyl-CoA production

It is quite astonishing that, for an unassuming oxidation reaction, a megadalton multienzyme assembly (∼4 MDa in bacteria; ∼10 MDa in eukaryotes) has persisted across billions of years of evolution (36) and has unique features related to its evolution across taxa (37). The complex is composed of three enzymes (E1, E2, E3) and a structural protein unique in eukaryotes (E3-binding protein, E3BP), in multiple copies. Cryo-EM has accelerated the structural investigation of the endogenous metabolon by deciphering the E3BP localization across taxa (21, 32, 38, 39, 40). E3BP, in lower eukaryotes, assumes a tetrahedral organization within the icosahedral E2 core, and it is likely to be sub-stoichiometric (38). Such stoichiometric variations have large consequences for the binding affinity for E3, manifesting as reduced E3 occupancy on E3BP or asymmetric localization of E3 on the PDHc outer surface, and therefore for the regulation of the entire link reaction. In mammals, instead, E3BP is localized in the icosahedral vertices of the E2, replacing E2 monomers (40), effectively reducing the PDHc core symmetry to tetrahedral (40). Still, outer subunits of the PDHc, composed of multiple copies of E1 and E3, and sometimes a few copies of regulatory kinase and phosphatase molecules, have not been resolved with the higher-order metabolon. Despite that, cryo-ET provided low-resolution information regarding their preferential orientations toward the PDHc core (41, 42, 43), which was sufficiently resolved due to its stability, suggesting potential spatial organization consistent with substrate channeling.

**Figure 3:**
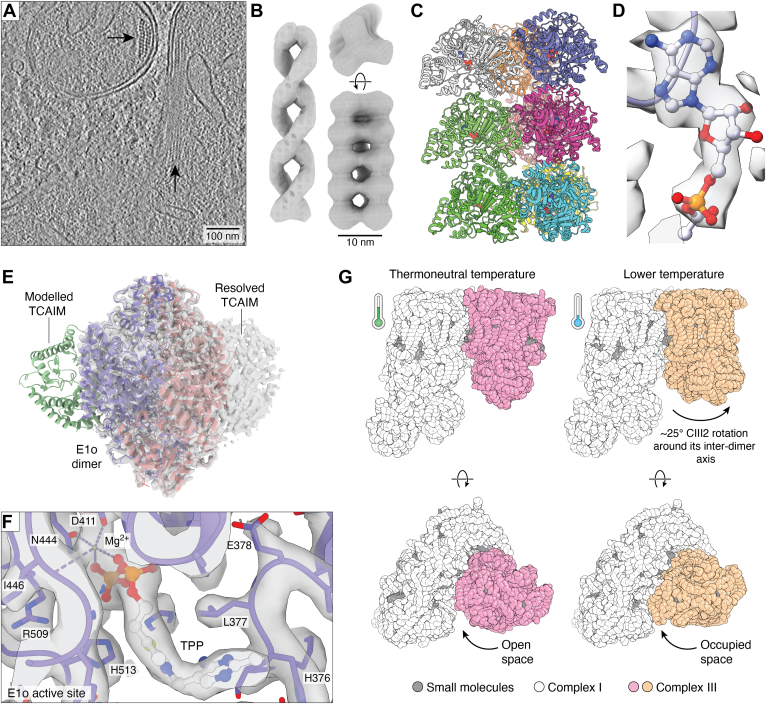
Cryo-EM insights into enzyme filaments and chaperone binding in acetyl-CoA metabolism (44), and temperature-dependent supercomplex conformational changes (93). (*A*) *in situ* tomogram of a yeast cell, showing various filaments within mitochondria (shown with *arrows*, EMD-19553). (*B*) subtomogram averages of filamentous structures derived from simplified cellular material, showing Ald4 (*left*) and Acs1 (*right*); resolutions are ∼44 and 18.3 Å, respectively (EMD-19552, EMD-19551). (*C*) Single-particle cryo-EM structure of Acs1 at 3.5 Å (EMD-19548, PDB 8RWJ), purified from meiotic yeast cells, showing identical subunit organization as the average in (*B*); (*D*) Identification of a purine derivative bound to Acs1. (*E*) Structure of the TCAIM-E1o complex (PDB: 8I0K) (58). The model does not superimpose on the deposited map (EMD-35042), as a rotation of the model is required. (*F*) The active site of E1o, in which thiamine pyrophosphate (TPP) along with the residues forming the cleft are resolved. (*G*) Temperature adaptations of the supercomplex I/III. The atomic models are represented with spheres corresponding to the main chain atoms (N, C, CA, CO, NH). An obvious conformation change is observed when comparing the molecular structures at thermoneutral and cold temperatures, with implications in electron transfer (93).

In addition to the canonical production of mitochondrial acetyl-CoA *via* the pyruvate dehydrogenase complex, recent cryo-EM studies have provided structural insights into an alternative acetate assimilation pathway in yeast mitochondria. This involves the potassium-activated aldehyde dehydrogenase Ald4 and the acetyl-CoA synthetase Acs1 (44). The two enzymes form linear multienzyme complexes that oxidize acetaldehyde to acetate (Ald4) and convert acetate to acetyl-CoA (Acs1) in meiotic cells and can be found in the nucleus, cytoplasm, and mitochondria. *In situ* cryo-EM showed that Ald4 forms filamentous structures; Acs1 trimers also form a canonical filament at regular intervals, both spanning the matrix space (Fig. 3, *A*–*D*). Filament structures are consistent with potential substrate channeling across protein subunits, and their interactions may facilitate acetyl-CoA production during fermentation. Conserved α-helical elements in both filaments mediate their association, and disruption of the filament interfaces reduced yeast growth on non-fermentable carbon sources *in vivo*. These findings underlie metabolically responsive scaffolds for acetate conversion and represent an important new paradigm of carbon flux regulation through mitochondrial metabolons (44).

### Krebs cycle

How acetyl-CoA is delivered to the Krebs cycle is still not detailed, while Krebs cycle intermediates are critical for cell function (45). It is conceivable that, at low concentrations (∼10–30 μM, resting cell state), acetyl-CoA slowly diffuses within the matrix, while at higher concentrations (∼50–200 μM in the fed state or high fatty acid oxidation and up to 300 μM in starvation or ketogenic state), acetyl-CoA directly inhibits PDHc and activates pyruvate carboxylase (45). The retrieval of native cell extracts has been a pioneering approach toward understanding the structure and function of Krebs cycle components. Interestingly, application of these methods (46) elucidated nine distinct mitochondrial complexes from 3.15 to 2.31 Å (FSC = 0.143). By utilizing advanced image processing (47), co-factors or substrates within calculated cryo-EM maps were resolved, and, in particular, FAD cofactor binding in three chemically distinct acyl-CoA dehydrogenases (46). While many of these findings were anticipated, such systems-based methods have the potential to retrieve surprising results. For instance, a soluble and catalytically active form of endogenous cytoplasmic myo-inositol phosphate synthase (MIPS) was resolved, revealing the presence of an acyclic sugar in its active site, an unexpected feature for a ground-state sugar substrate (48). Cryo-EM applied to increasingly heterogeneous specimens allows simultaneous characterization of distinct enzyme states, and such methodology can structurally probe different mitochondrial states and how the Krebs cycle may adapt.

In this direction, it is also shown that purified native mitochondrial membranes from mycobacteria embed the Krebs cycle enzyme malate:quinone oxidoreductase and are attached to the supercomplex formed by respiratory complexes III and IV (49). The finding holds significance for anti-mycobacterial strategies, given that multiple therapeutics act directly on this pathway. Overall, transient Krebs cycle interactions may be amenable to future structural characterization by cryo-EM, perhaps in combination with biochemical methods (50). For example, two-oxoglutarate dehydrogenase complexes (OGDHc) from various organisms are shown to contain multiple protein species, elucidated by cross-linking mass spectrometry (51, 52), and the community is now starting to structurally resolve fragments of this metabolon in different species (52, 53, 54, 55, 56, 57).

Recent structural and functional studies have revealed a layer of post-translational regulation of the E1 component of OGDHc (E1_o_), mediated by the mitochondrial DNAJC co-chaperone TCAIM (T cell activation inhibitor in mitochondria) (58). TCAIM specifically binds native, folded E1_o_ *via* defined β-repeat motifs, without altering its apo conformation, as shown by a high-resolution cryo-EM structure (Fig. 3, *E* and *F*) (note that the deposited structure file does not directly fit in the cryo-EM map after simply downloading the map and the model in a visualization software). Overall, binding in the mature protein contrasts classical heat shock proteins that act broadly on unfolded polypeptides. The interaction promotes E1_o_ degradation, reducing E1_o_ activity and consistency with a shift toward reductive glutamine carboxylation. TCAIM overexpression resulted in decreased respiration and OGDHc activity. TCAIM is, therefore, suggested to function as a unique type III DNAJC protein that regulates mitochondrial proteostasis by specifically downregulating a functional enzyme and, potentially, its entire metabolon.

Another study utilized the citrate synthase enzyme, a Krebs cycle enzyme, which constitutes the first example of a natural protein forming a fractal architecture (59). The authors showed that citrate synthase from the unicellular cyanobacterium *Synechococcus elongatus* assembled into Sierpiński triangles with a hierarchical geometry, reporting the first protein-based fractal architecture. This fractal form of citrate synthase has reduced enzymatic activity. However, its emergence highlights how complex supramolecular architectures can evolve and acquire regulatory potential and could well have implications in understanding mesoscale macromolecular organization.

### β-Oxidation of fatty acids and glutaminolysis

β-oxidation of fatty acids is the principal energy-yielding process in organisms ranging from bacteria to humans. In humans, more than 25 enzymes and specific transport proteins are involved in β-oxidation, and of these, most are known to be associated with human disease as inborn errors of metabolism (60). Enzymes progressively cleave fatty acid thioesters until complete degradation of the fatty acid in a process yielding acetyl-CoA, which enters the Krebs cycle. Although for short- and medium-chain fatty acids, a series of four independent enzymes perform the oxidation, for longer ones, an actual metabolon is formed. Here, the first reaction is carried out by the very long chain acyl-CoA dehydrogenase (VLCAD), and the next three β-oxidation reactions are performed by a single protein, the trifunctional protein (TFP). Recent cryo-EM studies elucidated the architecture of TFP at moderate resolution (61), resolving both an α_2_β_2_ tetrameric and an α_4_β_4_ octameric state. TFP is anchored in the extensively curved inner mitochondrial membrane. The subsequent crystallographic structure, together with the cryo-EM structure, elucidates the involvement of previously unknown membrane-submerged amphipathic α-helices of its β subunits in stabilizing its membrane-bound organization (62). Interdomain communication channels across subunits point to lipid-exposed interfaces that are consistent with TFP’s membrane association. Considering ongoing rapid cryo-EM advances, it is very likely that, during the next few years, the entire structure will be resolved at high resolution, delineating exact mechanistic details for β-oxidation.

Cryo-EM studies have also shed light on acetyl-CoA carboxylases (ACCs), key regulators of fatty acid metabolism that catalyze the ATP-dependent carboxylation of acetyl-CoA to malonyl-CoA, the first and rate-limiting step in fatty acid biosynthesis. In humans, ACC1 is cytosolic, while variant two localizes to the outer mitochondrial membrane, where it modulates β-oxidation by generating malonyl-CoA, an effective β-oxidation inhibitor. Initial ACC1 cryo-EM structures showed filamentation for the first time (63). Subsequent cryo-EM structures (2.55–2.73 Å (FSC = 0.143)) revealed multiple filamentous states of ACC1 (64), including a previously unidentified substrate-bound inactive filament and an active citrate-induced filament, suggesting structural regulation where domain rearrangements are associated with catalytic competence. Biotin was found to occupy a site distal to the acetyl-CoA binding site in the inactive filament, suggesting a resting state that prevents catalysis. Filamentation was also driven by p*H* changes, defining allostery as a regulator for subunit binding and catalytic activity.

Beyond fatty acid metabolism, recent cryo-EM studies are establishing glutaminase (GLS) as an example of mitochondrial enzyme filamentation. GLS filaments show that activation is induced by phosphate (P_i_) binding at the tetramer interface of the enzyme, which stabilizes the flexible activation loop and reorganizes the catalytic site (65, 66). Tetramers then assemble into helical filaments through lateral stacking (65, 66, 67). Mechanistically, therefore, filamentation stabilizes key regulatory elements of the enzyme. The resolved cryo-EM structures combined with functional assays show that the activation loop and a mobile domain region become ordered upon filamentation, locking the substrates (66, 67). In addition, mutations disrupting filament interfaces or preventing activation loop stabilization impair multimerization, demonstrating that such enzyme architecture is functionally important.

*In situ* cryo-ET revealed that GLS filaments form dense, aligned bundles within mitochondria under glutamine deprivation, validating the in-cell presence of the filaments observed *in vitro* (65). These filament-containing mitochondria exhibit elongation and resistance to fission and are associated with reduced mitophagy. Therefore, GLS filamentation should contribute to stress adaptation (65). These studies demonstrate how modern structural biology can probe complex enzyme architectures, which are becoming increasingly relevant (68), and bridge molecular structure with both metabolic regulation and mitochondrial ultrastructure.

### Oxidative phosphorylation (OXPHOS)

ETC complexes (I-IV) reside in the inner mitochondrial membrane, and their function relies on the precise arrangement and dynamics of their architecture, serving ATP synthesis (complex V). A detailed mechanistic understanding of Complex I utilizing cryo-EM was obtained 6 years ago (69). Its analytical structure–function correlations are also being elucidated (70, 71), and its evolutionary perspective through structural data was reported (72). Cryo-EM studies of mitochondrial membranes have been well-established (73), and can now provide a high-resolution view across ETC complexes (74). In addition, structural analysis of assembly pathways, specifically for Complex I, is reviewed (75). Complex I assembly incorporates at least 25 assembly factors, for which only a minority have been structurally characterized, *e.g.*, NDUFAF2 (76), NDUFAF1 and CIA84 (77).

Next, Complex II, also reviewed (78), was resolved by cryo-EM at ∼2.9 Å (FSC = 0.143) directly from human embryonic kidney (HEK) 293F by adding an affinity tag on one of its subunits (79). Besides its central role in disease (78), Complex II is implicated in the two primary metabolic pathways for generating ATP. It can function in either aerobic metabolism (termed succinate-ubiquinone reductase or succinate dehydrogenase) or anaerobic metabolism (termed quinol-fumarate reductase or fumarate reductase), therefore participating in both Krebs cycle and OXPHOS pathways. The cryo-EM structure revealed a conserved mushroom-shaped complex of four different subunits, as well as key redox-active cofactors, including flavin, iron-sulfur clusters, and heme groups. These were resolved in an arrangement ensuring efficient electron transfer. It was also shown that their proximity allows rapid electron flow. Furthermore, combined spectroscopic evidence confirmed the presence of ubiquinone, therefore proposing an entire model for electron flow (79).

Cytochrome *bc*_*1*_, also known as Complex III, has also seen extensive cryo-EM analysis in recent years (80). Novel structures include higher-order respiratory complexes (80) as well as its bacterial equivalent (81, 82). Similar progress has occurred for the human cytochrome *c* oxidase (Complex IV) (83), which is now known to be a monomer and not a homodimer (83). Proximity of ETC complexes was revealed by cryo-EM and has been analyzed in human cells (84), yeast (https://www.pnas.org/doi/epdf/10.1073/pnas.2021157118), plants (85), algae (86), and protists (87). A detailed review of how cryo-EM impacted the structural study of ETC complexes, their supercomplexes, and their intermediates can be found elsewhere (https://academic.oup.com/mam/article/31/1/ozae089/7762046?login=true).

ATP synthesis is the final step of aerobic respiration and is catalyzed by ATP synthase, a rotary enzyme complex also embedded in the inner membrane. ATP synthase is extremely abundant in the inner membrane, and, usually, *in situ* structural characterization of mitochondria directly recognizes its shape (88). An extensive review on the cryo-EM progress in the structure-function analysis of both ATP synthase and Complex I is available (89), which also discusses general mechanisms of ATP synthase dimerization identified across taxa. In these V-shaped homodimers in cristae membranes, a transmembrane subunit was shown to contain four long membrane-embedded α-helices arranged as two hairpins, tilted by ∼70° relative to the ATP synthase monomer (90). This arrangement is unusual for membrane proteins, but it is functionally essential as a conserved charged amino acid pair lies at subunit interfaces that couple proton translocation to molecular rotation. These and other cryo-EM data are approaching a dynamic structural description of ATP synthesis (at least for *Polytomella* sp. (https://www.science.org/doi/10.1126/science.aaw9128), and to a lesser extent for human cells (91)) and, in the near future, of complete OXPHOS. Observations such as the presence of multiple sites of cytochrome *c* oxidase on respirasomes, resolved at lower resolution (92), can guide current and future cryo-EM research to analyze OXPHOS supercomplexes and their higher-order assemblies at resolutions in which chemical insights can be derived.

Of notable importance is the visualization of structural adaptations and variations of ETC complexes to temperature variations (93). Specifically, this study resolved and analyzed 36 different cryo-EM maps, corresponding to temperature variations and defined subunits that alter their conformations and stoichiometries. The authors uncovered a new conformation forming at lower temperatures (4 °C *versus* 28 °C) in which the interface between Complex III_2_ and Complex I is modified. This is a consequence of a Complex III_2_ 25^o^ rotation around its own inter-dimer axis, perpendicular to Complex I (93) (Fig. 3*G*). Such adaptation is interesting beyond mitochondrial biochemistry, considering structural responses to physical-chemical and environmental changes.

### Mitochondrial calcium uniporter (MCU) complex, MRS2 Mg^2+^ channel, and LETM1 Ca^2+^/H^+^ antiport mechanism

In addition to substrate availability and redox status, mitochondrial energy metabolism is acutely regulated by calcium uptake through the mitochondrial calcium uniporter (MCU) complex. This complex’s structure–function relationship has been elucidated by cryo-EM at 3.7 Å (FSC = 0.143) (94). MCU is a tetramer, which selectively recognizes calcium through its central pore and facilitates most of the regulated Ca^2+^ uptake into the mitochondrial matrix. Human MCU was also solved in association with EMRE, a single-pass transmembrane protein required for functional MCU channels, at 3.8 Å resolution (FSC = 0.143) (95). The MCU–EMRE complex showed a dimer of tetrameric pore assemblies with EMRE subunits interfacing near the juxtamembrane loop and coiled-coil domains. This architecture allows EMRE-dependent gating, which is present in all metazoa. The holocomplex was determined by cryo-EM (96), including two more regulator proteins (MICU1 and MICU2). These findings illustrated the mechanisms of its calcium-dependent regulation in the presence of high (activated) or low (inhibited) Ca^2+^. These structures support a model in which under resting Ca^2+^, the MICU1/2 heterodimer caps the MCU pore like a gatekeeper, occluding ion flow. Upon elevated cytosolic Ca^2+^, conformational changes in MICU1/2 relieve the block and permit Ca^2+^ entry. Complementary fungal structures (*e.g. Neosartorya fischeri* MCU at 3.8 Å (FSC = 0.143)) (97) show a homotetramer whose pore-forming motif rings establish the selectivity filter as well, highlighting the conserved architecture of the channel.

Recent cryo-EM studies also elucidated a mitochondrial Mg^2+^ channel (mitochondrial RNA splicing two protein, MRS2). The human MRS2 cryo-EM structure (∼2.9–3.1 Å, FSC = 0.143) (98) revealed a pentameric inner mitochondrial membrane channel with a central pore defined by a conserved GMN motif and multiple ion-binding sites. Functional analyses also showed that MRS2 behaves as a Ca^2+^-regulated, nonselective cation channel permeable to Mg^2+^, Ca^2+^, Na^+^ and K^+^, indicating divergence from prokaryotic homologues. In parallel, cryo-EM structures of eukaryotic Mrs2 from *Chaetomium thermophilum* (*C. thermophilum*) were reported at 2.7 Å (FSC = 0.143, closed state) and ∼3.2 Å (FSC = 0.143, open state). These structures showed that Mg^2+^ binding stabilizes a symmetric, closed state, whereas Mg^2+^ depletion promotes channel opening (99). Both studies converge in reporting a conserved pentameric architecture of MRS2 and demonstrate that Mg^2+^ transport is governed by ligand-dependent gating mechanisms while also revealing species-specific differences in channel regulation.

LETM1 (Leucine Zipper and EF-Hand Containing Transmembrane Protein 1) functions as a mitochondrial Ca^2+^/H^+^ exchanger (100), and it was found to physically interact with TMBIM5 (also named mitochondrial morphology and cristae structure 1 (MICS1)) (101). Although high-resolution models are not yet available, negative staining analyses of LETM1 oligomers, reconstituted in proteoliposomes, demonstrate pH-dependent conformation changes of a hexameric complex that supports activity (102). These oligomeric assemblies may further regulate H^+^-dependent Ca^2+^ transport, consistent with LETM1’s regulatory role. Functionally, mitochondria lacking LETM1 display disrupted ionic balances, cristae swelling, and altered morphology (103). LETM1 expression triggers inward membrane curvature in proteoliposomes, and specific alanine substitutions prevent morphogenesis and functional complementation in yeast mutants (103). These analyses are consistent with a model in which LETM1 oligomerizes to form a p*H*-sensitive Ca^2+^/H^+^ antiporter, physically binds MICS1, and links mitochondrial bioenergetics and morphology.

Another inner mitochondrial entity, currently being mapped, is the mitochondrial permeability transition pore (mPTP). Its composition and nature are currently under debate (104, 105, 106). mPTP allows exchange of solutes across cristae and is regulated by cyclophilin D and Ca^2+^ to increase inner membrane permeability, thereby allowing passage of small molecules (<1.5 kDa) (107). The pore may arise from rare, Ca^2+^-dependent conformational changes in either the ATP synthase (potentially at the dimer interface) or from the adenine nucleotide translocase (108), possibly acting independently or in tandem in a hypothesized multiprotein complex (107). Another view is that mPTP is not in any way related to ATP synthase, that it is not a “channel,” but a less specific pore phenomenon that may not have a molecular basis in a regulated protein pore (106). A combination of molecular, biochemical, structural, and computational research on mPTP, across species, is therefore essential to resolve current views on its origin.

### Ketone body synthesis and monocarboxylate transport

Specialized compounds that contain ketone groups produced from fatty acids, dubbed “ketone bodies”, are important for cell bioenergetics, specifically during fasting or ketogenic states, and are synthesized in liver cells in a process called ketogenesis. Extrahepatic tissues, such as the brain, heart, and muscle, degrade ketone bodies in a process dubbed ketolysis. Both of these specialized pathways reside at the interface of the inner mitochondrial membrane and the matrix. For most involved enzymes, crystal structures of their overexpressed single-protein counterparts exist, but how the pathways are organized is elusive, or whether higher-order enzymatic architectures are adopted during either ketolytic or ketogenic pathways remains to be determined. Nonetheless, cryo-EM structures of involved transporters are now increasingly available. Monomeric monocarboxylate transporters (MCTs), specifically MCT1 (SLC16A1) and MCT2 (SLC16A7), are important for the import and export of ketone bodies, especially β-hydroxybutyrate and acetoacetate. Both MCT1 (109) and MCT2 (110) structures were resolved; MCT1, in particular, was determined bound to substrates and inhibitors at resolutions of 3.0 to 3.3 Å (FSC = 0.143) (109). However, note that MCT1 and MCT2 localize at the plasma membrane and possibly at the outer mitochondrial membrane, but the identity of the transporter(s) responsible for ketone body translocation across the inner mitochondrial membrane remains unresolved.

Overall, these recent studies in mitochondrial energy conversion collectively suggest that enzymatic reactions may be organized through transient macromolecular assemblies that adapt to metabolic demands. Cryo-EM is expected to further illuminate such mechanisms by revealing membrane association(s) and higher-order organization(s) consistent with substrate transfer and coordination across pathways. Importantly, many of these architectures emerge most clearly in endogenous or minimally perturbed systems, highlighting the importance of physiological context for their formation and regulation.

## Biosynthesis pathways

### Heme and ubiquinol biosynthesis

Heme b (Fe-PPIX) is an essential but cytotoxic cofactor synthesized on the matrix side of the mitochondrial inner membrane, where its production, trafficking, and conversion into hemes c, o, and a are localized. Hemes are utilized by several complexes (111), such as the ETC (112), hemoglobin, eNOS, and cytochrome P450 (CYP), and their dysregulation has been recognized in various cancers (113). The enzymatic steps of heme biosynthesis are well characterized (114). However, structural data for participating enzymes and their interactions are limited. Specifically, heme biosynthesis involves eight enzymatic steps, with the first and last three steps occurring in the mitochondria (115), and the intermediate steps in the cytosol. Crystal structures exist for the soluble enzymes, but mechanisms of heme mobilization and export are now being communicated at the structural level. Cryo-EM data describe high-resolution structures of mitochondrial ABC transporters ABCB6 (116, 117, 118, 119, 120) (also in its full length (121)), the ABCB7 transporter (122), and functionally related transporters from bacteria (123). ABCB6, linked to porphyrin uptake, binds cadmium *via* thiol peptides, having roles in both detoxification and porphyrin trafficking (118). ABCB7, involved in iron-sulfur cluster transport, displays an inward-facing conformation with bound ATP analogs (122), and structural mapping of disease mutations provides molecular insights into enzymopathies, *i.e.,* X-linked sideroblastic anemia.

Parallels between the biosynthesis of heme and coenzyme Q (CoQ) can be derived (124, 125), both performed at the inner mitochondrial membrane. Both pathways require coordination of multi-step reactions linking toxic intermediates and rely on complex transport systems that are poorly understood. Cryo-EM studies reveal CoQ complexes that may organize into a dynamic metabolon (complex Q). Notably, the structure of the COQ7-COQ9 subcomplex (126) suggests lipid extraction and presentation as important steps in CoQ maturation. These concepts are reminiscent of proposed substrate channeling in heme synthesis, and also resemble existing concepts explaining CoQ enzyme activity, such as spatial regulation, membrane association, and redox-sensitive interactions. These advances point to a broader theme in mitochondrial biochemistry, focusing on how hydrophobic cofactors are metabolized in organelle-spanning networks for signaling and redox balance.

### Iron–sulfur cluster assembly

Iron–sulfur (Fe-S) clusters assemble in mitochondria after the extraction of sulfur from cysteine (127). The first step of mitochondrial iron–sulfur cluster (ISC) biosynthesis is mediated by the *de novo* ISC assembly complex in the mitochondrial matrix, composed of five key proteins: the cysteine desulfurase NFS1, accessory proteins ISD11 and ACP, the scaffold protein ISCU, and the essential allosteric activator FXN (128). A low-resolution cryo-EM structure helped establish the overall organization (129). Cryo-EM analysis at 3.2 Å resolution (FSC = 0.143) elucidated the structure of the human complex (NFS1-ISD11-ACP-ISCU-FXN), dubbed SDAUF. It is a symmetric heterodecamer in which FXN bridges the interface between two NFS1 subunits and one ISCU molecule (130). FXN binding is proposed to stimulate NFS1 enzymatic activity by displacing the inhibitory Zn^2+^ ion from the scaffold ISCU, thereby exposing NFS1 catalytic residues for sulfur transfer. FXN also stabilizes key loop conformations in NFS1 and ISCU for sulfur delivery and cluster assembly. Mutations in FXN that disrupt these interfaces are linked to the pathology of diseases, such as the neurodegenerative Friedreich’s ataxia.

**Figure 4:**
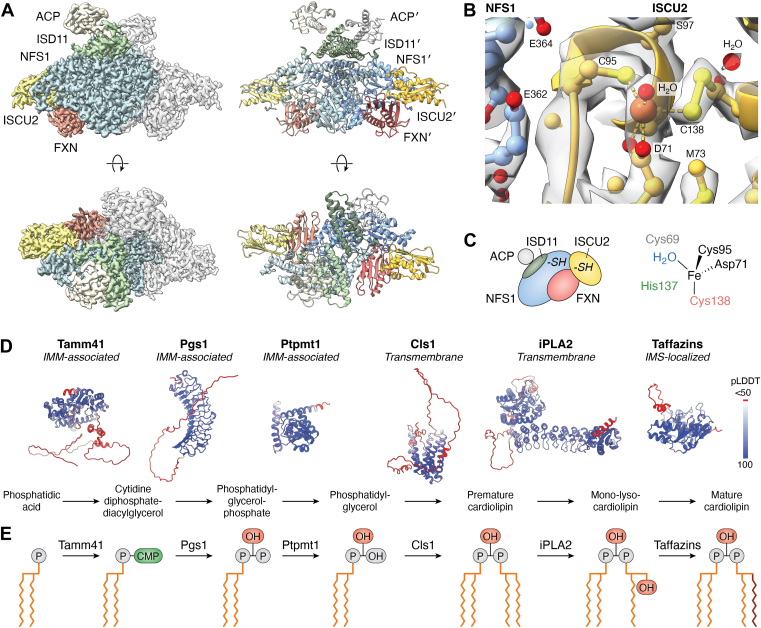
**Structural insights into cofactor and cardiolipin metabolism.** (*A*-*C*) The structure of the heterodecameric NFS1-ISD11-ACP-ISCU-FXN complex (131). (*A*) The high-resolution 2.49 Å structure during persulfide transfer (persulfide on ISCU2). Mapping of cryo-EM map (on the *left*) and atomic model (cartoon representation, on the *right*) is shown. (*B*) Fitting of the catalytic site to the cryo-EM map. Catalytic residues are shown, along with resolved water molecules. (*C*) Schematic representation of the complex (on the *left*), where the distal Cys sites between NFS1 and ISCU2 is shown. On the *right*, the described catalytic intermediate from the cryo-EM map, where the Cys residue from NFS1 is in the distal state, and organization of the iron is performed by catalytic residues in ISCU2 and an embedded, resolved water molecule. (*D*-*E*) Major enzymes acting within the pathways are highlighted (panel (*D*)), with their biochemical function mapped below (panel (*E*)). AlphaFold models from humans are shown, retrieved from the AlphaFold database (https://alphafold.ebi.ac.uk/).

Other, higher resolution structures of the system are being communicated (131, 132). Both are novel because they provide the first high-resolution cryo-EM analysis of the human Fe^2+^-bound ISC complex with ferredoxin-2 (FDX2) under anaerobic, near-physiological conditions. Note that FDX2 is the crucial electron donor for Fe-S cluster biogenesis. In one of those, the ISC complex includes two distinct FDX2 conformations, *i.e.,* “proximal” and “distal,” that are proposed to regulate electron transfer to the ISCU2 Fe-S cluster assembly site (131) (Fig. 4, *A*–*C*). Here, FDX2 and FXN compete for overlapping binding sites on NFS1, addressing ambiguities about their interactions. The overall architecture and a resolved state under anaerobic conditions are shown in Figure 4.

The next steps in Fe-S cluster metabolism have not yet been structurally analyzed: Biochemically, we know that this cluster is then transferred, with the help of chaperones, to carrier proteins. Some [2Fe-2S] clusters are then converted into [4Fe-4S] clusters by another ISC complex (133), where FDX2 acts again as the electron donor. Specialized assembly factors catalyze the final insertion of these clusters into target mitochondrial proteins. A sulfur-containing intermediate is also exported to the cytosol to support Fe-S protein maturation outside the mitochondria *via* a partially known pathway (128).

### Steroid hormone biosynthesis, cardiolipin, and other phospholipid biosynthesis

Cholesterol transfer across mitochondrial membranes is the first step in steroid biosynthesis. A cytochrome family member, CYP11A1, localizes in the inner mitochondrial membrane and converts cholesterol to pregnenolone. This rate-limiting step requires two electron-transfer partners, the ferredoxin reductase (FDXR), and the adrenodoxin (FDX1) (134). Currently, no cryo-EM structures of these partners are resolved, but their available models are derived from X-ray crystallography, including a CYP11A1-FDX1 complex (135) and structures of individual redox proteins. Therefore, for this step, spatial coordination of cholesterol cleavage and electron transfer has not been visualized at high resolution. In addition, homologs of the small mitochondrial translocator protein, mediating cholesterol import into mitochondria, have been reported by NMR (136) and X-ray crystallography (137), identifying cholesterol-binding pockets and effects of sequence changes. Similarly, the StAR protein, which delivers cholesterol to the inner membrane, lacks a high-resolution structure of its full-length form due to its intrinsic disorder, with only its C-ter ordered domain resolved *via* X-ray crystallography (138) or small peptides recognized by 14-3-3 scaffold proteins (139). Thus, while cryo-EM is instrumental for resolving the structural biology of many mitochondrial systems, its accuracy in visualizing steroidogenesis is currently limited.

Phospholipid biosynthesis within mitochondria is also structurally elusive regarding enzyme-enzyme interactions. Briefly, key lipids are produced and remodeled, such as phosphatidic acid (PA), cardiolipin (CL), phosphatidylethanolamine (PE), and phosphatidylglycerol (PG) (140). Phospholipid synthesis begins in the endoplasmic reticulum (ER), but mitochondria localize dedicated enzymes for the final steps and remodeling (141). Mitochondria import phosphatidylserine (PS) from the ER and convert it to PE *via* the mitochondrial phosphatidylserine decarboxylase (PISD), an inner membrane-anchored enzyme. PISD’s catalytic domain shares homology with other decarboxylases, but no full-length or cryo-EM structure of (parts of) these mitochondrial pathways exists to date. The CL mitochondrial biochemistry has been reviewed (142). Briefly, conversion of PA to CDP-diacylglycerol (CDP-DAG) is performed by CDP-diacylglycerol synthase 1 (CDS1), which mostly localizes to the ER (143), and instead, its functional homologue Tamm41 localizes to the inner membrane (143). CDP-DAG then acts as a precursor for PG synthase (PGS1) and downstream for CL synthase (CLS1), after a phosphatase acting on the lipid (PTPMT1).

Following its initial synthesis, CL undergoes a maturation process involving remodeling of its acyl chains. This includes the action of calcium-independent phospholipase A_2_, which introduces hydroxyl groups by removing specific acyl chains. Then, phospholipid transacylases such as tafazzins (TAZ), localized in the mitochondrial intermembrane space, mediate the reacylation step, incorporating unsaturated fatty acids to produce mature CL (Fig. 4, *D* and *E*). AlphaFold models of major enzymes in CL synthesis and maturation, as well as their underlying accuracies, are shown in Figure 4*D*. AlphaFold is a protein structure prediction approach that infers three-dimensional models from amino acid sequences. In contrast to cryo-EM, where structures are experimentally determined, AlphaFold models provide hypotheses that can guide and accelerate subsequent structural studies. These models will very likely pave the way to resolve them with cryo-EM in various metabolic states in the near future. This is crucial as mutations in the TAZ gene impair this remodeling, leading to Barth syndrome (reviewed in (144)). Interestingly, TAZ is also involved in Complex I maturation, at least in orthologous complexes, and may participate in early assembly intermediates by providing mature CL at the location of membrane protein assembly (77).

### Trafficking of biosynthesized mitochondrial components: The MICOS system

A key player in lipid trafficking is the mitochondrial contact site and cristae organizing system (MICOS), which cooperates with the ER-mitochondria encounter structure (ERMES) in yeast or PDZD8/mitofusin-based tethers in mammals to support phospholipid exchange at membrane contact sites (145) and has been suitably reviewed (146). New technologies in cryo-EM have allowed direct, in-cell visualization of these contact sites (147). Using cryo-correlative light and electron microscopy, subtomogram averaging, and molecular modeling, ERMES was shown to form ∼25 discrete bridge-like complexes per contact site, arranged in a flexible zig-zag configuration. This configuration spans the cytosol and connects the ER to the outer mitochondrial membrane through a continuous structural conduit that is proposed to support lipid transfer between membranes. The organization, length variability, and clustering of bridges suggest a regulated supramolecular architecture, a higher-order transport scaffold, where ERMES may act less as a shuttle and more as a stable membrane-anchored bridge.

Structural and interactome analyses are also resolving how the Sorting and Assembly Machinery (SAM) and the MICOS complex form a supramolecular assembly known as the Mitochondrial Intermembrane Space Bridging (MIB) complex, while biochemical analysis (148) currently accelerates in-cell cryo-ET studies of the MIB complex (149). This physical coupling is mediated by the interaction of the Mic60 MICOS component from the inner membrane and the Sam50 component of SAM from the outer membrane, forming a trans-compartmental scaffold. It is proposed (150), based on resolved cryo-EM structures from bacteria or yeast (151, 152, 153), that the β-barrel protein Sam50, once thought to function primarily as a chaperone in assembling β-barrels at the mitochondrial outer membrane, also participates in the MIB complex across species. Functionally, MIB complex integrity is essential to maintain crista junction architecture and phospholipid distribution, and disruption of either Mic60-Sam50 contacts or their higher-order assemblies leads to cristae disorder and impaired respiratory supercomplex assembly (150).

Next, compositional analysis of MICOS is currently being actively pursued: MICOS comprises two subcomplexes, namely Mic60-Mic19-Mic25 and Mic10-Mic12-Mic26-Mic27. For subcomplex I, Mic60 is known to have coiled-coil domains (resolved by X-ray crystallography (154)), while Mic19 regulates MICOS curvature-generating activity (155). For subcomplex II, Mic10 knockout produces the most severe CJ defects (equivalent to those from Mic60), effectively abolishing crista junctions (156). Mic10 oligomerizes into hairpin-based scaffolds that bend membranes independently of Mic60 (157), while Mic12 connects the two subcomplexes to form the holo-MICOS complex (158).

In mammals, mitochondria-ER contact sites (MERCs) are organized by a tethering complex involving PDZD8 (159), Miro1/2 (160), and mitofusin 2 (Mfn2) (145, 161). However, no cryo-EM or cryo-ET studies have yet been reported that resolve the architecture of this complex at sufficient resolution to delineate its individual components. Consequently, progress in this field has relied on the identification of interacting biomolecular partners. These contact sites suggest that mitochondrial function is influenced by intermembrane coordination, a principle that is further exhibited within the organelle itself at the level of cristae organization. Current evidence supports a model in which the architecture of cristae depends on the cooperative action of MICOS, ATP synthase dimers, and the dynamin-related GTPase OPA1 (162, 163). The OPA1 ortholog in fungi, Mgm1, was studied in the thermophilic fungus *C*. *thermophilum* with structural and cryo-ET analyses (164) showing that Mgm1 assembles into bent tetramers and helical filaments on curved membranes.

ATP synthase dimers bend the inner membrane into tight curves, while MICOS anchors the junctional rim and tethers to the outer membrane *via* SAM. Meanwhile, OPA1, resolved in cryo-EM models as a membrane-bound dynamin-like helical assembly, modulates junction diameter and inner membrane fusion (165, 166). Disruption of any component (*e.g.,* ATP synthase dimerization, Mic60 loss, or OPA1 cleavage) may lead to cristae fragmentation or ballooning. OPA1, MICOS, and ATP synthase are therefore best viewed as components of an integrated cristae-shaping system, but more research in the composition of the various contact site complexes is required to proceed to higher-resolution structural studies.

Collectively, these works show that mitochondrial biosynthesis depends on coordinated spatial organization across membranes, contact sites, and transient assembly states. Cryo-EM uncovers lipid remodeling and intermembrane trafficking processes from a structural perspective while also making clear that major gaps remain for pathways dominated by flexible, low-abundance, or partially membrane-embedded components. Thus, biosynthetic pathways currently illustrate both the power and the present boundaries of cryo-EM. While they expose regulated architectural principles, they also define a major frontier for a closer-to-life structural analysis.

## Protein, amino acid, and nitrogen metabolism

Mitochondria act as a hub for the metabolism of nitrogen and amino acids, including critical steps in the urea cycle, amino acid catabolism, and one-carbon metabolism. These processes are tightly compartmentalized, involving membrane-associated enzyme systems.

### Urea cycle

The urea cycle is the primary mechanism of nitrogen excretion in ureotelic organisms and begins in the matrix with the formation of carbamoyl phosphate and citrulline. Two enzymes, carbamoyl phosphate synthetase I (CPS1) and ornithine transcarbamylase (OTC), catalyze the initial reactions and localize at the inner membrane (167), while the rest of the cycle is performed in the cytosol. Both mitochondrial proteins have been resolved by crystallography (*e.g.,* the 1500-residue monomer of CPS1 (168) and the homotrimer of OTC (169)). It is of note that OTC can form either trimers or hexamers (170), and its oligomerization state is not currently understood. Their structures share homology with those of the trifunctional multi-domain enzyme involved in the first three steps of pyrimidine biosynthesis, for which low-resolution structures from EM data are currently available (171). Based on current literature, the long-standing hypothesis from the 1980s and 1990s that the urea cycle enzymes may form a compartmentalized metabolon (172) remains plausible.

### Amino acid degradation pathways

Mitochondria degrade several amino acids *via* OXPHOS and transamination, producing intermediates that feed into the Krebs cycle, ketogenesis, or gluconeogenesis. The branched-chain α-keto acid dehydrogenase complex (BCKDc), in particular, remains a major target for architectural elucidation because it shares organizational features with PDHc and OGDHc. An extensive review of the family (173) covers advances in the study of the two-oxoadipate dehydrogenase complex (OADHc) as well. OADHc plays a critical role in degrading lysine, hydroxylysine, and tryptophan. Cryo-EM studies of those complexes, including the glycine cleavage complex, although theoretically feasible, are currently lacking, mainly because of their size, undefined component composition and stoichiometry, and in-cell scarcity: BCKDHc, for example, is a rare biomolecular assembly to be retrieved in cell extracts and its endogenous purification or *in silico* classification is very challenging (32, 52). Notably, their architecture is highly complex and only reconstitutions of functional, truncated α-keto acid dehydrogenases from bacteria are currently feasible, specifically PDHc (174).

Next, cryo-EM analysis of glutamate dehydrogenase (GDH) (175) showed both initial and steady-state phases of catalysis are composed of multiple metastable conformations. These findings challenge the classical view of a single enzyme-cofactor-substrate complex. NAD-domain motions, such as hinge-bending and shearing transitions were identified and correspond to different ligand-binding and catalytic states, with structural transitions modulated by hydration and local side-chain conformation changes. These results support the view that the Michaelis complex comprises an ensemble of interconverting conformers, consistent with a complex energy landscape underlined by conformational heterogeneity. GDH also serves as a standard for cryo-EM, besides apoferritin (176), resolved to 1.8 Å resolution (FSC = 0.143). In addition, its known filamentous state, which has an as yet unknown function, has been discovered by EM in the past. Accordingly, future high-resolution structural studies may clarify whether GDH multimerization contributes to enzymatic catalysis or cellular homeostasis (68). Other enzymes have limited cryo-EM data; however, the proline dehydrogenase-P5C dehydrogenase metabolon has enough data (177) to be revisited for high-resolution analysis.

### One-carbon metabolism: structural framework of the mitochondrial folate cycle

The mitochondrial folate cycle contributes one-carbon units for nucleotide synthesis, methylation reactions, and redox balance (178). Key enzymes include the serine hydroxymethyltransferase 2 (SHMT2), the methylenetetrahydrofolate dehydrogenase 2 (MTHFD2), and the formyltransferase-cyclohydrolase (MTHFD1L), all of which are now partially or fully resolved by structural methods, or have available, relatively confident AlphaFold models. In particular, human SHMT2 has been deposited at 2.9 Å resolution (FSC = 0.143) using cryo-EM (PDB ID: 8QI7), but has not been analyzed or described in the literature yet. SHMT2 was discovered to be implicated in immune signaling, forming a specific complex with an isopeptidase (dubbed BRISC), which performs K63-specific deubiquitylation (179). This study revealed the 3.8 Å (FSC = 0.143) structure of the BRISC-SHMT2 complex. The structure showed that only the inactive SHMT2 dimer, and not the higher-order tetramer, which is stabilized by pyridoxal phosphate, sterically inhibits the deubiquitylase active site of BRISC. This interaction links one-carbon metabolism to inflammatory signaling, as intracellular pyridoxal phosphate levels regulate BRISC-SHMT2 binding and downstream type I interferon responses. These results support a metabolite-sensitive mechanism through which SHMT2 may influence inflammatory signaling independently of its canonical metabolic role.

Research findings described above clarify biochemical evidence for metabolon formation in the urea cycle and α-keto acid dehydrogenase systems, but structural validation at the level of intact assemblies is still lacking. In contrast, cryo-EM analyses of enzymes such as glutamate dehydrogenase and SHMT2 show discrete conformational and metabolite-sensitive interaction states, indicating that regulation occurs through defined structural transitions. However, their integration into larger metabolic networks is still unknown. To more effectively structurally analyze these pathways, growth conditions must be optimized to enrich specific metabolic states, thereby increasing the abundance of target assemblies. This is particularly important to overcome a key limitation of cryo-EM applied to endogenous material, namely the difficulty of detecting low-abundance, repetitive signatures of biomolecular complexes and their interactors within heterogeneous cellular material.

## Transport and import system

### The pyruvate transporter

**Figure 5:**
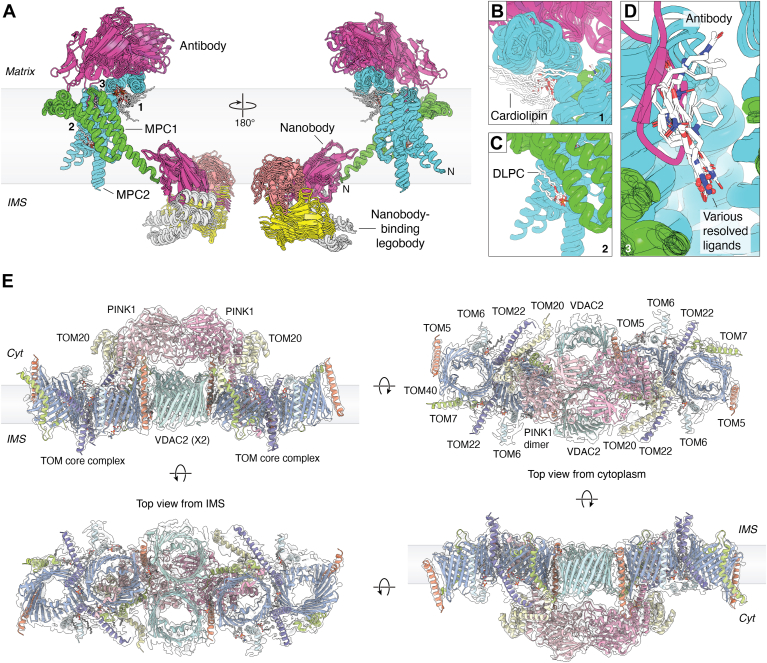
**Organizational principles of selected mitochondrial transport machineries.** (*A*-*D*) Superposition of all MPC structures reported (180, 181, 182, 183). (*A*) MPC is a transmembrane dimer, composed of monomers having three transmembrane α-helices each, and three regions of interest are highlighted (1,2, and 3). (*B*) Region (1); Cardiolipin molecules are frequently bound, and have similar orientations in respect to the transmembrane α-helices. (*C*) Region (2); Here, only one structure showed clear density to resolve a lipid (dilauroylphosphatidylcholine, DLPC). (*D*) Region (3); This defines the catalytic site of MPC, where the antibody occupies the cavity in the unbound state and substrate-bound structures show conserved ligand positioning. MPCs are shown in cartoon representation and color-code is as follows: MPC1 corresponds to *green*, MPC2 to cyan, antibodies and nanobodies used to *magenta*, and the three chains of the nanobody-binding legobody to *white*, *yellow*, and *pink*, respectively. Lipids and small molecules are shown in sticks with standard atom color-coding. (*E*) cryo-EM structure (EMDB-48083, PDB: 9EIH) of the outer membrane-embedded TOM supercomplex together with the PTEN-induced kinase 1 (PINK1) is organized as a dimer. The analyzed interfaces in this work provided the basis for signaling during mitophagy (199).

The mitochondrial pyruvate carrier (MPC) is a heterodimeric complex (typically MPC1/MPC2 or MPC1L/MPC2) that imports pyruvate. 17 structures of the previously unresolved pyruvate transporter*, i.e.,* the human mitochondrial pyruvate carrier (MPC), were published across four publications within the first half of 2025 (180, 181, 182, 183). All structures were determined using specific antibodies to increase contrast in cryo-EM acquisitions. Most antibodies recognized the matrix side of the MPC, while nanobodies with an additional nanobody-binding legobody (an engineered extension or fusion domain added to a nanobody that provides an additional binding interface) bound to the N-ter of the intermembrane space side (182). Superposition of all structures showed a rather rigid MPC1/MPC2 complex (Fig. 5*A*). These structures correspond to key transport states, *i.e.,* outward-open, occluded, and inward-open, and support an alternating-access mechanism mediated by pseudo-symmetric rigid-body displacements of transmembrane α-helices and lipid binding (Fig. 5, *B* and *C*). A central, water-filled substrate cavity was identified (Fig. 5*D*), where two residues, K49 (MPC2) and H86 (MPC1L), form the core of a conserved, pH-sensitive pyruvate-binding site. The *N*-terminal amphipathic helices reside in the intermembrane space, contradicting earlier MPC topological models (Fig. 5*A*). Moreover, inhibitor-bound structures show that chemically diverse compounds all bind to the same conserved interfacial pocket (Fig. 5*D*), engaging key residues such as K49 and H86 through ionic and polar interactions. These insights clarify MPC topology, conformational changes, and inhibitor binding, and provide a structural framework for future MPC-targeted therapeutic development (184) for cancer, neurodegeneration, metabolic diseases, and steatotic liver disorders.

### Mitochondrial protein import: TIM, TOM, and the elusive TIM/TOM supercomplexes

Mitochondrial protein import is not only highly regulated (185), but also highly responsive to stress (186). It relies on conserved multi-subunit translocases that guide nuclear-encoded proteins across the organelle’s double membrane (11). This is also important to understand, as only 13 proteins are encoded in human mitochondria, all essential subunits of OXPHOS complexes. Most mitochondrial proteins are transferred through translocase complexes, which include the translocase of the outer mitochondrial membrane (TOM) and the translocase of the inner mitochondrial membrane (TIM23). For both membrane complexes, cryo-EM has advanced our molecular understanding (187) while in-depth reviews on structure-function correlations of those translocase assemblies are being communicated (188). Cryo-EM structures of the TOM complex in yeast (189) and human cells (190) reveal a dimeric architecture formed by two Tom40 β-barrel channels, each fully folded with 19 β strands, flanked by small α-helical subunits and bridged by the Tom22 central receptor. Tom40 forms the protein-conducting pores, each shaped by an internal regulatory α-helix, and Tom22 aids substrate recognition and dimer stabilization.

Other proteins support oligomerization, *i.e*., tetramer formation, regulate assembly and guide precursors at the IMS interface. A bound phosphatidylcholine lipid stabilizes the dimer, and electrostatic mapping suggests distinct substrate paths through the negatively charged Tom40 lumen. A tetrameric TOM arrangement was also resolved by cryo-EM (191). This may regulate cooperative import, supporting a model in which Tom proteins recruit precursors that are translocated through Tom40 by gating and charge-based steering. A cryo-EM structure of a TOM complex was also reported from genome-edited *C. thermophilum*, where substrates, its conserved stoichiometry, and distinct conformations were described (192).

β-barrel proteins like Tom40 are not fully matured upon import by their mature TOM complex forms. Instead, their insertion into the outer mitochondrial membrane is completed by the SAM complex (see above; reviewed in (193)). Cryo-EM structures of the SAM complex with Tom40 (152) from human cells, but also with other binders (194), provide a structural basis for translocation events. Specifically, the SAM-Tom40 complex exhibits a well-defined stoichiometry, consisting of one copy each of the three Sam proteins and Tom40. Sam50 forms a 16-stranded β-barrel, has a partially open lateral gate, and an *N-terminal* soluble domain, while the other two Sam proteins cap the complex on the cytosolic side. Stepwise addition of TOM subunits preserves the core architecture, maintaining near-identical conformations pre- and post-substrate release. The final TOM subunit, Tom22, clashes sterically with SAM, pointing to a mature complex that is released through structural incompatibility, consistent with a β-barrel switching model.

The TIM23 complex, responsible for importing presequence-containing proteins across the inner membrane, has been resolved *via* cryo-EM in both overexpressed and endogenous states (195). These structures support a model in which Tim17, and not Tim23, forms the main translocation cavity and includes both conserved acidic residues and a lateral opening toward the membrane. Tim23, previously proposed to form the aqueous pore, acts primarily as a structural scaffold. Functional and cross-linking studies support this architecture, showing substrate interaction with Tim17 and lateral gate control by the small subunit Mgr2. This arrangement allows full translocation into the matrix and membrane insertion without requiring a continuous channel. The import motor PAM, including mtHsp70, generates ATP-dependent pulling forces but remains unresolved at high resolution. These insights refine long-standing biochemical models, although they may represent only one state within a dynamic transport cycle. This, in principle, necessitates more cryo-EM data regarding different steps in mitochondrial protein translocation (196).

Finally, the TIM22 complex mediates the insertion of hydrophobic metabolite carriers, including SLC25 family members, into the inner membrane. Cross-linking data (197) and cryo-EM structures of the human TIM22 (198) revealed conformational flexibility and multiple interaction sites for the hexameric architecture formed by the component subunits. The structure, in particular, resolved at 3.7 Å (FSC = 0.143), showed that the overexpressed construct was 440 kDa and consisted of Tim22, Tim29, AGK (acylglycerol kinase), and two hexameric chaperones: Tim9/10a and Tim9/10a/10b. The core transmembrane region is formed by four α-helices of Tim22 that shape a lipid-exposed cavity with conserved residues, and is flanked by the single transmembrane domains of Tim29 and AGK. Tim29 appears to act as a structural scaffold, linking the chaperones and AGK to the translocation core, and AGK contributes both to membrane anchoring and structural interactions within the assembly. All these translocases are in proximity within the double mitochondrial membrane, and recent studies have begun to report their higher-order assemblies, such as TOM signaling complexes captured at a stalled state (199) (Fig. 5*E*).

Another example is the substrate-engaged TOM-TIM23 metabolon, which was first studied integratively at ∼10 Å (200), and then briefly described in a short communication (at ∼4.4 Å (FSC = 0.143) and with cross-linking at ∼3.6 Å (FSC = 0.143), but here, most high-resolution signal was derived from the TOM complex (201). The TOM-TIM23 metabolon is tilted at angles of ∼30 to 50° relative to each other, and their centers are separated by ∼50 Å (200). The complex was also captured with a translocating polypeptide, but still, TIM was, again, at low resolution (202). The TIM-TOM metabolon, considering available information, will be an excellent example in the near future to perform integrative structural biochemistry studies to link its nanoscale and mesoscale architectures.

### Cryo-EM advances in mitochondrial carrier proteins

The mitochondrial carrier system, responsible for metabolite exchange across the inner membrane, is dominated by the 53-member SLC25 family of transporters, which mostly operate *via* a gated rocker-switch mechanism (203), but not always (204). This mechanism is well-known and involves binding of the substrate to an open gate on one of its sides; then, the gate closes, isolating the substrate. Afterwards, the rocker-switch, which corresponds to a conformation change of the receptor, promotes rotation of the substrate, flipping the orientation, and finally, the opposite gate opens, releasing it. Of course, in order to follow such a mechanism, most receptors, if not all, are monomeric (205), and therefore, long-range allosteric effects are greatly reduced.

Besides the pyruvate transporter described above, a revolution in their high-resolution cryo-EM analysis is ongoing, which is excellently covered in the literature (206, 207). Uncoupling protein 1 (UCP1, SLC25A7), a key mediator of non-shivering thermogenesis in brown adipose tissue, dissipates the protonmotive force to generate heat, has also been reviewed (208), and will serve as an example. In brief, cryo-EM structures of human UCP1 in its GTP-bound, ATP-bound, and unliganded forms (209) have revealed a canonical SLC25 carrier fold, stabilized by CLs and locked in a proton-impermeable state by purine nucleotides. These nucleotides bind a central cavity framed by conserved residues that include an arginine triplet (R84, R183, R277), which interacts with phosphate groups of the substrates. These carrier-ligand interactions stabilize a closed conformation of the complex through salt-bridge networks. A proposed activation mechanism by fatty acids, however, remains elusive. Although 2,4-dinitrophenol binds weakly to UCP1, it does not induce structural changes or proton permeation, therefore does not mimic physiological activation (210). Sequence conservation, conformational flexibility, and comparison with related SLC25 transporters suggest that UCP1 may retain the capacity to cycle between matrix- and cytoplasm-facing states, essential for proton transport. These structural insights provide a basis for UCP1 activation and may inform anti-obesity therapeutic development (211).

### Shuttle systems: The carnitine shuttle, NADH/NAD^+^ balance, and CoA transport

The carnitine shuttle system, critical for long-chain fatty acid import into the mitochondrial matrix, involves CPT1 at the outer membrane (carnitine palmitoyltransferase 1), CACT (carnitine/acylcarnitine translocase) in the inner membrane, and CPT2 on the matrix side. While structural models of CACT (SLC25A20) are inferred from SLC25 family homologs and a relatively accurate AlphaFold model, high-resolution cryo-EM structures of the human proteins are lacking. Biochemical data suggest a substrate-induced conformational switch that alternates acyl-carnitine across the inner membrane in a 1:1 exchange with free carnitine while the proteins are assembled in an outer membrane fatty acid transfer metabolon (212).

In parallel, two shuttle systems are critical for maintaining the NADH/NAD^+^ redox balance across the impermeable inner membrane: the malate-aspartate shuttle and the glycerol-3-phosphate shuttle. The former operates through coordinated transport of malate, α-ketoglutarate, glutamate, and aspartate *via* SLC25 carriers (*e.g.*, SLC25A11 and SLC25A12), none of which have yet been resolved by cryo-EM, and only the soluble *N-ter* domain of SLC25A12 was determined in different functional states by X-ray crystallography more than a decade ago (213). AlphaFold models, however, show promising accuracy again and can be used for structure-based discovery of endogenous carriers from mitochondria. The glycerol-3-phosphate shuttle, active mostly in muscle and brain, uses a mitochondrial glycerol-3-phosphate dehydrogenase (mGPDH) anchored in the inner membrane that channels electrons directly to ubiquinone. A full cryo-EM structure of mGPDH is not yet available, but homologous information suggests that its FAD-binding domain is oriented toward the intermembrane space, with proximity to complex III for direct ubiquinone access.

Finally, among the SLC25 mitochondrial carrier family, SLC25A42 mediates the import of CoA into the matrix, fueling acetyl-CoA synthesis, fatty acid oxidation, and Krebs cycle entry, while embedded mutations cause myopathies in humans (214). Its structure is also not resolved, and homology is only shared with few resolved ADP/ATP carrier proteins, with less than 30% of sequence identity (215, 216, 217). The AlphaFold prediction has partial confidence as reflected by lower pLDDT scores, *i.e.*, a per-residue confidence metric indicating the reliability of the modeled structure, for its *N-ter* transmembrane helix points to potential conformation changes and mechanistic relevance that can only be uncovered by structural interrogation. Overall, structural studies for carrier systems are underrepresented, mainly due to their small size. However, the example of the pyruvate transporter can inspire upcoming structural research in other shuttle systems, where its structural knowledge dramatically expanded within 6 months (184).

Conclusively, mitochondrial transport is now transitioning from inferred mechanisms to structurally defined assemblies, but with uneven resolution across systems. MPC provides a near-complete structural framework for substrate recognition and alternating-access transport (but *in vitro*), whereas protein import machineries increasingly show partially resolved translocation pathways from endogenous material but remain structurally and therefore functionally incomplete. In parallel, most SLC25 carriers and shuttle systems are still represented by isolated states or predicted models, limiting mechanistic interpretation of metabolite exchange. This contrast highlights a central challenge: resolving transient, membrane-embedded, and multi-component transport processes across functional states, particularly for lower-mass carriers and dynamic supercomplexes.

## Mitochondrial gene expression

### mtDNA replication and maintenance

Mitochondrial DNA replication has traditionally been analyzed through biochemical models (218); mutations on the mitochondrial DNA polymerase show reduced lifespan and premature onset of ageing phenotypes in mice (219), and there is still new knowledge elucidated for the mitochondrial DNA composition, *e.g.*, an additional promoter sequence in human cells (220), great gene copy variation across tissues (221), mTOR regulation *via* transcriptional control of mitochondrial oxidative functions (222), and its role in mammalian fertility (223). Cryo-EM is also charting this biochemical pathway. The structure of the eukaryotic mitochondrial replisome is yet elusive, but its constituents are known, and current single-particle analysis (SPA) methods can probe aspects of its dynamics (224, 225).

DNA polymerase γ (Pol γ) is the single replicative mtDNA polymerase which synthesizes both heavy and light strands. Therefore, it possesses 5′→3′ polymerase and 3′→5′ exonuclease proofreading activities, correcting mismatches and misincorporations. Pol γ functions within a processive complex, together with the TWNK helicase, and the mitochondrial single-strand DNA-binding protein. Following strand synthesis, it also degrades 3′ termini at nicks to prepare for ligation. Pol γ contributes to base excision repair too, catalyzing 5′-dRP lyase activity at apurinic/apyrimidinic sites, and therefore, repairs mtDNA without metal ions. Individual structures of Pol γ and TWNK are resolved with cryo-EM, but not of mtSSB yet (mutations of which have drastic effects on ssDNA compaction ability and binding dynamics) (226).

**Figure 6:**
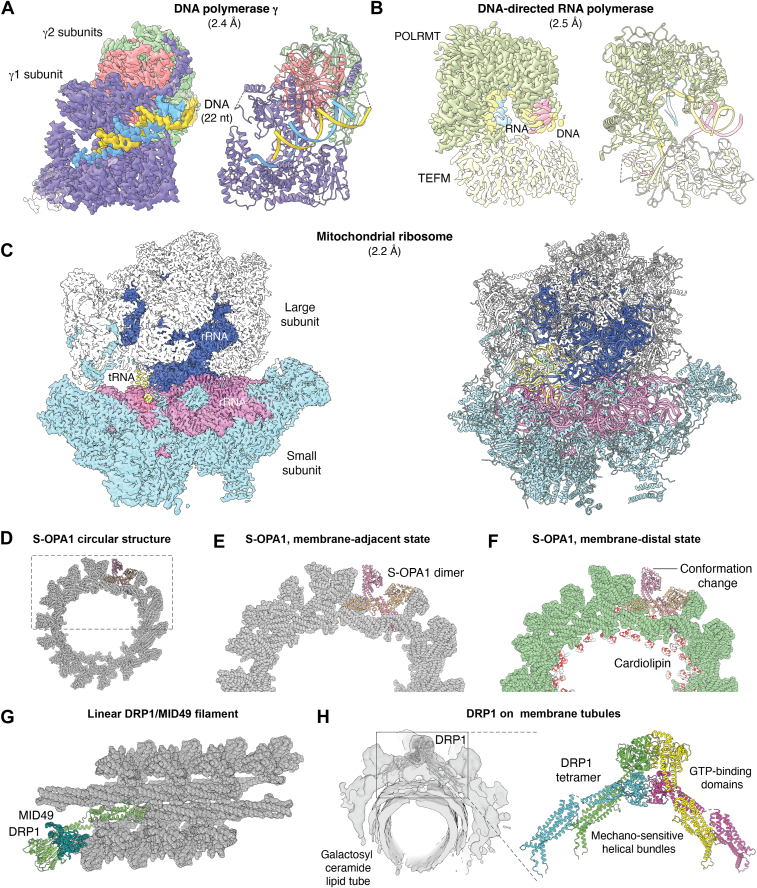
**Structural data underlying DNA replication, transcription, and translation, and higher-order mitochondrial dynamics.** (*A*) DNA polymerase γ with DNA (EMD-42150, PDB: 8UDL). (*B*) DNA-directed RNA polymerase during transcription (EMD-44448, PDB: 9BDC). (*C*) The mitochondrial ribosome (EMD-13980, PDB: 7QI4). (*D*-*H*) S-OPA1 and DRP1 higher-order structures on membranes. (*D*) S-OPA1 structure, forming a circular architecture (EMD-26977, PDB: 8CT1) (165); (*E*) is a higher magnification view of (*D*), where the S-OPA1 dimer polymerizes into those higher-order structures; (*F*) When S-OPA is in a distal site, conformation changes are observed that propagate across the circular structure, as well as tight interactions with cardiolipin (EMD-26984, PDB: 8CT9). (*G*) Filament-forming DRP1-MID49 dimers, and assembling into arrays (EMD-8874, PDB: 5WP9) (268). (*H*) When galactosyl ceramide lipids are used, DRP1 decorates the tubular lipid scaffold as a tetramer (EMD-43045, PDB: 8V8T).

Specifically, Pol γ is a dimer of two subunit (γ-1, γ-2) with 26 communicated structures of different states (7 resolved by X-ray crystallography (227, 228, 229, 230) and 19 by cryo-EM (230, 231, 232, 233)). Interestingly, this is a case in which the achieved cryo-EM resolutions are, in general, higher than those obtained by crystallography, with the highest resolution reported for wild-type Pol γ bound to DNA (230) (2.37 Å (FSC = 0.143), FSC = 0.143) (Fig. 6*A*). Twinkle was also resolved by cryo-EM (234), albeit at lower resolutions, including a corresponding clinical variant. Its bound structure to DNA is only resolved in sea bass (235). Cryo-ET of mitochondria *in situ* has visualized replicating nucleoids (236); combined with superresolution microscopy, nucleoids are shown to be irregularly ellipsoidal in shape, 90 to 130 nm in diameter, and to form regardless of the mtDNA copy number. The mitochondrial transcription factor A (TFAM) compacts mtDNA through cross-strand bridging and cooperative binding, supporting the view that such compaction is important for regulating mtDNA accessibility during replication and transcription. In the near future, in-cell cryo-ET, studying lamellae derived from ion/plasma cryo-focused ion beam milling, and scanning electron microscopy (cryo-FIB/SEM), will cast light to such structures within mitochondria at ever increasing resolution.

### mtDNA transcription and translation

Mitochondrial gene expression, once thought to follow simplified bacterial principles, has now emerged as a structurally and mechanistically distinct system (237, 238, 239). Earlier X-ray crystallography studies elucidated the structure of mitochondrial DNA-directed RNA polymerase (240), its elongation complex (241), and its interactions with additional factors (242) and promoters (243). Cryo-EM has then resolved its complexes with inhibitors (244) as well as its higher-order states as a homodimer (245). This homodimer is formed in the presence of a non-coding 7S RNA molecule, consistent with a negative-feedback mechanism. Specific mutations in the polymerase abolish dimerization (245). Various conformations involved in substrate binding and selection were reported (246): Here, intermediate states that correspond to architectures of “entry” and “insertion” sites were captured. In the entry site, ATP interacts with conserved residues but remains spatially separated from the templating DNA base, allowing binding of rNTPs or dNTPs. Upon conformational change, the polymerase transitions to the insertion-site state, where the substrate is positioned for catalysis by forming Watson-Crick base-pairing with the template. This study also identified a “substrate rejection” state, where non-cognate nucleotides are expelled following an abortive attempt to engage the insertion site. These structures, especially high-resolution ones (Fig. 6*B*) underlie a functional model of induced fit and Brownian ratchet mechanisms, with implications for mapping mitochondrial gene expression and modelling the entire mitochondrial replication in the near future.

Mitochondrial translation and the mechanisms behind mitochondrial ribosome assembly are likewise being redefined and are subjects of many focused reviews (247, 248, 249, 250, 251). From the first seminal reports (252, 253), the field of structurally characterizing human mitochondrial translation has moved impressively forward. To date, more than 90 cryo-EM models of mitochondrial ribosome structures have been deposited in the PDB (www.pdb.org), with some of them achieving impressive resolutions in terms of the gold-standard Fourier shell correlation (*e.g.,* up to 2.2 Å (FSC = 0.143) (254, 255)) (Fig. 6*C*). Notably, the ribosome is organized at the mitochondrial membrane through recruitment by the Oxidase Assembly 1-Like (OXA1L) membrane protein, and the complex is resolved, albeit to lower resolutions (5–8 Å) (256).

At that resolution, modified rRNA residues and disulfide-linked or reduced cysteine residues are visible. Such resolutions are close to potentially identifying small molecules, and even partial hydration properties. This impressive compendium comprises translation and assembly intermediates. These, if put together in a field-specific perspective similar to the one published 4 years ago (247), a “molecular movie” of mitochondrial translation will be potentially achievable. This is also true for mitochondrial aminoacyl-tRNA synthetases, for which structural data are progressively enriched and is also the subject of field-specific reviews (251, 257, 258, 259). Despite this, their spatial architecture is not yet resolved, and is an active area of research (260, 261), where high-resolution tomographic methods are applied to decipher translation dynamics *in situ* (262).

Understanding of mitochondrial gene expression is progressing toward a structurally resolved, multi-step process, yet remains fragmented at the level of interacting assemblies. High-resolution cryo-EM has defined specific catalytic states and regulatory conformations of individual components (*i.e.*, for Pol γ, transcription complexes, and mitoribosomes). However, higher-order genetic organization, such as the mitochondrial replisome, nucleoids, and spatial coupling of transcription and translation, remains unresolved at a resolution where even protein components can be resolved. Bridging scales, from well-defined enzymatic intermediates to intact gene expression machineries within nucleoids remains an upcoming challenge for mitochondrial molecular biology research.

## Beyond “classic” mitochondrial metabolism

### Structural analysis of fusion, fission and mitophagy mechanisms

Cryo-EM and cryo-ET are expanding structural analysis of other critical regulatory systems of mitochondria, beyond core metabolism, biosynthesis, and gene expression. Mitochondrial dynamics, including fusion and fission, are now captured in increasing detail. Truncated structures of mitofusin-1 (263, 264) and mitofusin-2 (265) have been resolved by X-ray crystallography and support models in which GTP-dependent conformational changes mediate outer membrane fusion; however, their higher-order structures are currently elusive. This is not the case for OPA1, a dynamin-like GTPase, which mediates inner mitochondrial membrane fusion. OPA1 was deciphered at multiple conformations and higher-order states (165, 166) (https://doi.org/10.7554/eLife.50294). Structurally, OPA1 exists in two forms: the membrane-anchored long form (L-OPA1) and the soluble short form (S-OPA1), which results from proteolytic cleavage. Both isoforms are proposed to cooperate to mediate inner membrane fusion, during which OPA1 assembles into higher-order tubular structures. L-OPA1 may initiate membrane remodeling by recruiting S-OPA1 to form flexible, cylindrical scaffolds around the inner membrane. The process is driven by OPA1 dimerization and membrane-insertion of α-helices which interact with CL-enriched domains (166) (Fig. 6, *D*–*F*).

Structural data support a model in which OPA1 filaments assemble within the tubular inner membrane regions that correspond to crista junctions. This OPA1 activity can regulate inner membrane fusion and fine-tune cristae morphology. The equilibrium between L-OPA1 and S-OPA1 is important. Excess S-OPA1, especially following mitochondrial stress or depolarization, disrupts this balance and is associated with impaired fusion, fragmentation, and cristae disorganization. Structurally, changes in the L-/S-OPA1 ratio correlate with distinct cristae morphologies: Cristae are stacked and elongated with L-OPA1 abundance, but are irregular and spherical upon its depletion. Such insights were communicated by *in situ* cryo-ET at medium resolution (266). Conversely, dynamin-related protein 1-driven (DRP1) mitochondrial fission is now being structurally understood as a staged helical constriction process (Fig. 6, *G* and *H*), with high-resolution cryo-EM resolving its unbound (267) and receptor-bound states (268), the former being an autoinhibited dimer, and the latter promoting filamentation. Its assembly is also mediated, as in the case of OPA1, by CL interactions (269). Its low-resolution structure on lipid membranes was also reported (270).

Mitophagy and apoptotic clearance are also emerging for structural characterization. Cryo-EM of the PINK1 kinase (199, 271) structurally reveals its function as a membrane potential sensor. When mitochondria lose their membrane potential, PINK1 accumulates on the outer membrane and initiates a signaling cascade that flags the organelle for autophagic degradation. PINK1 is specifically bound to components of the TOM complex and localizes in proximity to two voltage-dependent anion channels (199) (Fig. 5*E*). These findings support that PINK1 becomes stalled during import under depolarizing conditions, promotes dimerization of TOM/TIM-associated assemblies, and participates in a signaling-competent complex that initiates mitophagy. Similar structures are expected to be communicated for Parkin as well, an E3 ubiquitin ligase participating in the next steps during mitophagy (272), as its correlated structure-function dynamics are now investigated by NMR spectroscopy (273). Parkin mutations are associated with mitochondrial dysfunction, leading to neuronal death in Parkinson's disease (274) and aberrant metabolism during tumorigenesis (https://doi.org/10.1172/JCI185838). Finally, during apoptosis, cytosolic BAX monomers translocate to the mitochondria and permeabilize the outer membrane, a process described by cryo-ET at medium resolution (https://elifesciences.org/articles/40712). BAX pores (275) show a strong variability of higher-order structures, forming linear, arc, polygonal, and ring polymers. Missense mutations in the resolved interfaces impair pore formation, supporting the view that polymorphic BAX multimers modulate membrane permeabilization.

### Proteostasis, redox mechanisms, and NAD^+^ maintenance

Mitochondrial-embedded proteostasis pathways are also now being described at high resolution. AAA^+^ proteases (276, 277) (https://www.science.org/doi/10.1126/science.aao0464) adopt conserved hexameric ring structures with substrate engagement coordinated across membrane-anchored or matrix-localized domains. Specialized types of these proteases have been linked to lipid signaling and mitochondrial proteome changes that resemble those reported in cancer patients (278). Another protease, LONP1, acquires distinct spiral staircase conformations within its ATPase and protease domains. Substrate engagement is required at both sites for proteolytic activation (277). Further studies resolved eight nucleotide-dependent conformational states, almost entirely describing its catalytic states (279). The ClpXP system, a two-component protease complex composed of the AAA+ chaperone ClpX and the peptidase ClpP, was also studied by cryo-EM (280): Here, the hexameric ClpX interacts with the stacked heptameric ClpP rings despite symmetry mismatch, forming a substrate translocation pathway for activating its protease function. Other mitochondrial chaperones, extensively studied through structural data, include TRAP1 (281) and HSP60 (282). Both are also being investigated by X-ray crystallography due to their high crystallization tendency. The above data illustrate that mitochondrial unfolded protein response (UPR^mt^) is facilitated through complex regulated structural transitions and cycles of stress-dependent inhibition.

Mitochondrial redox biology is currently also in focus: cryo-EM reconstructions of Complex I and III in distinct conformational and compositional states demonstrate that reactive oxygen species (ROS) generation is modulated by specific redox geometries and supercomplex architecture (283, 284), therefore arguing against the view that ROS are purely stochastic byproducts. Antioxidant enzymes, such as PRDX5 and NNT, are being structurally defined in various redox states, promoting the model of localized, structure-based ROS detoxification. Cryo-EM, therefore, begins to reveal spatial assemblies of antioxidant machinery that respond to ROS surges. Although α-keto acid dehydrogenases are critical for this (285, 286), accounting for ∼45% of the total mH_2_O_2_ formed by mitochondria, their highly complex structures prohibit specific structure-function analysis. Finally, mitochondrial NAD^+^ maintenance is gaining attention, specifically the role of NMNAT3 in matrix-localized NAD^+^ synthesis. Structural information is incomplete, and mostly corresponds to X-ray structures of its dimeric free form. Other NMNAT3 homologues, *e.g.*, NMNAT1, are also being resolved by cryo-EM, together with biomolecular interactions (287), but their involvement in mitochondrial NAD^+^ maintenance is not apparent, as they were found not to localize therein. NMNAT3 resides in the mitochondrial matrix, where it is proposed to sustain intramitochondrial NAD^+^ pools required for dehydrogenase activity and redox balance.

Beyond the systems detailed above, several emerging structural themes will enrich mitochondrial structural biology. Recent studies are beginning to define how mitochondrial protein complexes respond to reversible oxidative post-translational modifications (PTMs), such as cysteine sulfenylation (288) or lysine acetylation (289, 290), which fine-tune enzymatic activity and import specificity under metabolic or redox stress (291). Cryo-EM will be soon capturing such PTM-induced conformational shifts for protein complexes, which are highly important for regulating mitochondrial metabolism, *e.g.,* PDHc inhibition *via* phosphorylation (*i.e.*, as shown for the overexpressed thiamin-dependent pyruvate dehydrogenase (E1) component (292)). At a larger scale, mitochondrial contact sites extend beyond ER-mitochondria bridges, including lysosomal, peroxisomal, and even lipid droplet interfaces (293); however, structural data for these connections are sparse, but advances in cryo-ET and cryo-correlative methods, for which progress in high spatial precision is being performed (294), are bound to shed light on these transient organellar junctions. The mitochondrial ribosome quality control machinery, involving an ever-evolving list of important partners and cofactors (295, 296), has also begun to reveal stalled translation states as rescue mechanisms for mitochondrial proteostasis (297). These results integrate with ongoing structural insights into AAA+ proteases and chaperones to suggest a surveillance-based proteome logic within mitochondria.

Across taxa, cryo-EM comparisons of mitochondrial complexes, *e.g.*, OXPHOS stoichiometry, ATP synthase organization, and cristae-shaping modules, are revealing deep evolutionary adaptations linked to energetic demands (78, 148, 298, 299). At the translational level, cryo-EM is increasingly used to resolve drug-bound mitochondrial complexes, such as inhibitors of MPC (182), UCP1 (209), or ETC subunits (300), setting the stage for structure-guided therapies in cancer, neurodegeneration, and metabolic disease. Finally, conceptually novel organizational principles are being proposed, including condensate-like behavior (*e.g.*, in TFAM-mtDNA assemblies (301)) and stress-responsive biomolecular phase separation (302). These developments suggest that mitochondria have structurally defined regulatory layers that dynamically adapt to physiology, pathology, and therefore, therapeutic targeting, awaiting structural analysis in future cryo-EM studies.

The processes described in this section are very challenging to resolve and display notable plasticity. Cryo-EM has partially analyzed polymeric and higher-order states for key effectors (*e.g.*, OPA1, DRP1, and BAX), and multi-state catalytic cycles for AAA+ proteases and redox-active complexes. Nevertheless, upstream signaling pathways and inter-organelle coordination remain poorly studied, lacking resolution information. This highlights a critical gap in our understanding of organelle dynamics and emphasizes the need to expand research frontiers where cryo-EM-driven integrative structural biochemistry can be further utilized.

## Conclusions and perspectives

Recent advances in cryo-EM are voluminous, and only a fraction of those were covered in this review. An overview is shown in Figure 1, where major mitochondrial metabolic pathways are shown to be systematically enriched with structural data, covered in the sections above. Cryo-EM has illuminated molecular architectures behind nearly every mitochondrial subsystem, offering unprecedented insights into how structure governs metabolic function. From respiratory chain supercomplexes and ATP synthase dimers to protein import machineries and genome maintenance modules, mitochondrial biochemistry has been redefined through electromagnetic lenses. The emerging atlas of high-resolution models, openly available in the PDB (www.pdb.org), provides mechanistic insights and disease-relevant contexts, detailing the molecular bases of mitochondrial-related diseases. Cryo-EM is positioned as a central tool for decoding mitochondrial function in both physiological and pathological states. Although its equipment and infrastructure are very expensive, the enormous insights gained by cryo-EM cannot be underestimated simply because of its running costs. Indeed, cryo-EM continues to reveal the fundamentals of life in detail, which would have been unimaginable just 10 years ago (303, 304).

However, major frontiers remain to be explored. Our understanding is that structural data on ROS as signaling agents are just now emerging, but it is unknown how specific these effects are. Similarly, PTMs dynamically regulate mitochondrial activity, and cryo-EM is beginning to approach a clear understanding of their structural, and therefore, functional consequences. Mitoribosome-associated quality control mechanisms are currently being actively elucidated, but the correlated structural/cellular evidence linking them to translation surveillance and neurodegenerative disorders are yet to come. Despite its centrality in cellular metabolism, mitochondrial lipid biochemistry remains underexplored in terms of structural analysis. Enzymes involved in CL remodeling, fatty acid oxidation, and acyl-CoA trafficking are implicated in human disease, and future structural studies must focus on clarifying their molecular mechanisms and structural systems biology. Additionally, mitochondrial contact sites are functionally essential but structurally elusive, with cryo-ET being properly equipped to resolve those at ever-increasing resolutions. Calcium signaling, particularly through the MCU-MICU1/2 complex (305), now benefits from high-resolution reconstructions that reveal gating mechanisms and regulatory adaptation under variable ionic loads. However, such changes are rapid, and currently, no method exists at the required resolution to approximate the real-time component of such events with structural data.

Beyond these established systems, additional areas are evolving where structural insight is likely to be relevant, but challenging due to the highly irregular molecular nature of the biomolecules to be analyzed. Mitochondrial RNA granules, which are composed of LRPPRC, GRSF1, and related RNA-binding factors, underlie transcript stabilization and processing, but are very heterogeneous to be addressed at increasingly higher resolution. Mitochondrial-derived vesicles, which traffic damaged proteins and lipids to lysosomes or peroxisomes, currently lack molecular models but also represent a challenging, but underexplored axis of mitochondrial quality control. Another promising area is the emerging evidence for biomolecular condensates within mitochondria, and a review of those was communicated (302). These phenomena point to an organizational logic beyond membrane-bound compartments and simple biomolecular interactions. Across these lines, it is yet unknown if structural analysis of mPTP is even feasible.

Comparative structural biology also holds great potential: variations in respiratory complexes, ATP synthase architectures, and cristae morphology across taxa, from anaerobic protists to photosynthetic eukaryotes, offer insights into mitochondrial specialization. Such studies are being performed on photosystems, *e.g.*, (306, 307, 308, 309), and therefore, the mitochondrial community can be inspired. Cryo-EM is increasingly deployed in structure-guided drug discovery, visualizing mitochondrial targets bound to small-molecule inhibitors or metabolic modulators. These developments have potential for translational impact across cancers, metabolic syndromes, obesity, aging, and neurodegeneration, all phenotypes in which mitochondria undergo drastic changes. Together, all these fields for structural research highlight the versatility of cryo-EM not only in mapping mitochondrial structure, but in recovering deeper understanding of regulation and, potentially, therapeutics. Cryo-EM continues to push resolution boundaries and to expand into *in situ* and time-resolved measurements. Such developments will move the field closer to fully capture the organelle’s role as an adaptive metabolic superstructure.

The discoveries analyzed in this review reveal a modular and adaptive organization of the pathways. Importantly, these advances are often so accurate that the underlying chemistry of the biomolecules involved can be decoded. Cryo-EM allows functional interpretations of how structure encodes enzymatic efficiency and adaptive regulation. A central conceptual advance from cryo-EM is the realization that mitochondria are structurally dynamic organelles, populated by transient, multivalent, or condition-specific assemblies that often escape detection in classical reductionist models (*e.g.,* from overexpressed systems). Single-particle cryo-EM has begun to resolve some of these intermediates, but much of the future lies in approaches that preserve native molecular context. Cryo-ET, importantly, provides an emerging toolkit for visualizing mitochondrial structures *in situ*, within intact organelles and cells.

Complementing these *in situ* approaches, structural studies of mitochondrial fractions, cellular homogenates, and enriched extracts, now serve as a powerful middle ground. By applying cryo-EM directly to biochemically tractable preparations that retain partial complexity, such as mitochondrial lysates or mechanically sheared cell extracts, one can partially preserve native interactions and increase particle abundance, orientation diversity, and reconstruction quality. This hybrid level of complexity accelerates discovery of low-abundance assemblies, context-dependent stoichiometric variations, and multienzyme complexes, *i.e.*, metabolons, that are destabilized in traditional purification pipelines. In our subjective point of view and experience, these semi-native preparations detect previously unknown conformational states and resolve transient biomolecular components that are otherwise invisible in fully isolated or reconstituted systems (310, 311, 312). It is of note that around half of the discussed cryo-EM structures in this review have been isolated from endogenous sources (Fig. 1).

As all these modalities to probe mitochondrial structure converge (SPA; native cell extract structural biology; *in situ* structural analysis with cryo-ET) through advances in hardware and software for cryo-EM, the field moves closer to decoding the organizational principles of cells (313), and therefore, also for mitochondria, revealing embedded functions from resolutions allowing *de novo* atomistic model building to network-level communication. A key challenge ahead, definitely, is bridging the gap between atomistic models and cellular spatial context, particularly for dynamic, low-copy, or highly specialized complexes, and several recent ideas about creating real-time digital twins (314) may have unexpectedly successful applications in the future for elucidating simpler systems, such as organelles. The increasing use, but also, the responsible use, of machine learning, optimized sample preparation, and hybrid pipelines will be important. In this ever-evolving methodological landscape, cryo-EM is no longer just a structural tool; it is a system-level lens, across resolution regimes, through which mitochondrial biochemistry becomes accessible.

Overall, the research reviewed here underlies a conceptual shift in how mitochondrial biology is now perceived (as compared to, *e.g.*, a few years ago): An emerging model illustrates biochemical processes embedded within spatially organized and reconfigurable assemblies. Within this framework, conformational heterogeneity is an integral feature that regulates functional responses to substrates, cofactors and interactors, and at a higher level, to metabolic and environmental parameters. At the same time, growing emphasis on endogenous and *in situ* structural approaches indeed shows that physiological context is becoming integral in understanding the function of any mitochondrial biomolecule. These emerging principles are consistent with a systems-level view of mitochondrial metabolism where traditionally perceived stable molecular entities are deciphered in coordinated and dynamic higher-order assembly states.

## Conflict of interest

The authors declare that they have no conflicts of interest with the contents of this article.
